# Amorphous silicon intrinsic photomixing detector for optical ranging

**DOI:** 10.1038/s44172-023-00137-5

**Published:** 2023-12-04

**Authors:** Andreas Bablich, Maurice Müller, Rainer Bornemann, Nils Marrenbach, Paul Kienitz, Peter Haring Bolívar

**Affiliations:** University of Siegen – School of Science and Technology, Hölderlinstr. 3, 57068 Siegen, NRW Germany

**Keywords:** Optical sensors, Engineering

## Abstract

Today’s optical range finders or 3D imagers suffer from significant drawbacks and do not allow to combine performance (sensitivity, precision) with simplicity, and scalability enabling very-large scale integration with minimum footprint. Here, we present the amorphous silicon Intrinsic Photomixing Detector (IPD) for direct and highly sensitive optical envelope mixing. The ability to generate a photocurrent that is proportional to the nonlinear mixing of two optical modulation envelope functions enables high performance Time-of-Flight optical ranging at low light levels down to $\sim \!0.1\,{{{{{\rm{mW}}}}}}{{{{{\rm{cm}}}}}}^{-2}$ at $444\,{{{{{\rm{nm}}}}}}$. The IPD exceeds MHz bandwidth, covers a broad distance detection range from $10\,{{{{{\rm{cm}}}}}}$ to $101\,{{{{{\rm{m}}}}}}$, and achieves a mean depth resolution below $44\,{{{{{\rm{mm}}}}}}$ for distances up to $25\,{{{{{\rm{m}}}}}}$. The IPD paves the way towards simple but high-performance photodetectors that allow for very-large scale integration on top of silicon or flexible electronics at low costs with pixel fill factors up to 100%.

## Abstract (web summary)

Bablich and colleagues report an Intrinsic Photomixing Detector (IPD), a cost-effective amorphous silicon device, enabling high-bandwidth, long-distance, and high accuracy Time-of-Flight optical ranging even in low light conditions. The device allows for large-scale integration on silicon or flexible platforms.

## Introduction

Optical ranging and three-dimensional (3D) imaging are already key sensing techniques and established in a huge variety of application fields, e.g. in the automotive sector for autonomous driving^1^, driver assistance and security systems^2^. 3D imaging plays an important role to generate comprehensive datasets serving as input for deep learning^3^, artificial intelligence (AI)^4^, or neuronal networks^5^. Considering the Internet of Things in the industrial sector, it is possible to develop autonomous and enhanced robot systems reaching a new level of efficiency in production and enabling high-performance product manufacturing based on 3D image/scene recognition in combination with cyberphysical systems^6^. Further fields of 3D imaging applications cover the medical sector for optical-assisted diagnostics and surgery^7^, the consumer market (smart devices) as well as virtual-/augmented reality (VR/AR)^8^ including the Metaverse^9^. The huge application bandwidth for 3D imaging systems goes along with a wide spreading range of specification requirements including achievable operating range, depth and pixel resolution, speed, technological compatibility, system size, mobile usage and acceptable prizing, necessitating the establishment of fundamentally different sensing approaches and techniques. Prominent and reliable 3D imaging systems are based either on triangulation or on the Time-of-Flight (ToF) principle; each approach suffering from specific drawbacks. Although ToF image sensors and systems can enable long-range detection exceeding hundreds of meters^10^, pixel fill factors and achievable depth resolutions are limited. Due to the high speed of light, timing the round-trip time is difficult and depth resolutions of common solid-state 3D imagers limited to a few millimeter (cf. Table 1). Precision may further be pushed down to 70 μm going along with massive technological and measurement efforts^11^.

Alternative triangulation-based systems obtain a strongly reduced operating range up to a few meters only, but often achieve depth resolutions in the μm-range^12,13^. To achieve such a precision, complex algorithms and post-imaging processes are required to extract the depth information, impeding a fast image acquisition. In addition, triangulation-based systems invariably require a significant spacing between the light source and the detector which—at a certain degree—prohibits a very-large-scale integration, e.g. in compact smart devices.

Today’s light detection and ranging (LiDAR) systems often rely on the direct ToF approach and utilize avalanche photodiodes (APD) or single photon avalanche photodiodes (SPAD) for high precision time stamp detection. Such photodiodes are operated in Geiger mode at very high reverse bias voltages of <<−12 V^14,15^ and require highly complex post-detection circuity to (i) erase and reset the desired avalanche effect and (ii) protect the sensor from irreversible destruction^16,17^. In result, pixel fill factors of state-of-the-art solid-state SPAD 3D camera systems are limited to $13.4 \%$^15^. Emerging nanophotonic based LiDAR systems utilize metasurfaces as spatial light modulators (SLM) for beam steering in combination with highly sensitive APDs and achieve distance detection up to $10\,{{{\rm{m}}}}$ with a depth resolution of $4\,{cm}$ in a single-point measurement^18^. Indirect ToF represents a second mayor principle in this field. Here, the time or, in case of amplitude modulated light, a phase delay is measured. These techniques often rely on heterodyning processes. Prominent devices, e.g. the photonic mixing device (PMD), utilize a smart but rather complex device architecture and signal post-processing to enable unambiguous ToF distance determination^19^. The PMD is an electro-optical mixer and correlator that intricately mixes an amplitude- modulated reference signal with a second phase-shifted signal from the reflected scene. This principle invariably requires an elaborate signal post-processing, the so called cross-correlation, for unambiguous distance determination. In solid-state 3D imagers using PMD technology, pixel fill factors stagnate at about $22 \%$ due to extensive electrical circuitry^20^. Besides scaling limitations, the detection range of such PMD imagers only achieves a few meters^20^ due to the limited sensitivity of the sensor and laser eye safety issues. A rather new ToF approach that adapts principles from radar technology is the frequency modulated continuous wave (FMCW) method. Although the robustness of FMCW towards stray-light rejection and parasitic background illumination is advantageous, the laser and detection unit integration effort is comparatively high and expensive^21^. In summary, today’s 3D imagers suffer from significant drawbacks and do not allow to combine performance (sensitivity, precision) with simplicity, and scalability enabling very-large scale integration with minimum footprint.

Besides conventional bulk photodetector architectures, novel high-speed nonlinear photodetectors based on atomically thin graphene have been reported and achieve envelope ($\equiv$ intensity) frequency mixing of an electrical with one optical^22,23^ or two optical^24^ signals at GHz modulation bandwidth. Along with lacks in scalability and reproducibility, the 2D material-based approach actually suffers from low conversion efficiencies requiring irradiances in the range of MWcm^−2^ ^22^.

Amorphous hydrogenated silicon (a-Si:H) and its alloys, a well-established material class that relies on a mature low-temperature plasma enhanced chemical vapor deposition technology (PECVD), becomes more and more important in a variety of application fields. In optical communication and logic systems, such materials enable wavelength conversion at high data rates due to nonlinear optical processes^25^. In a-Si:H waveguides, optical (N)AND logic gate operation in the time domain at GHz frequencies has already been demonstrated utilizing four-wave mixing Bragg scattering; a third-order nonlinear process that requires high intensity laser illumination^26^. In a-Si:H field-optimized p-i-n photodetectors and solar cells, it was found that collection efficiencies can exceed values of long-term optimized crystalline silicon photodiodes that utilize photogating in the visible range^27–29^. Compared to conventional high-temperature silicon manufacturing, the mature thin-film deposition technology of a-Si:H allows for ubiquitous sensor integration on top of read-out electronics at low temperatures and costs with pixel fill factors up to $100 \%$ ^30,31^.

Due to simplicity and handling, in first demo tests only large pixel samples of 1.6 mm × 1.8 mm on rigid substrates have been evaluated. The aspect of flexible electronics and wearables as demonstrated previously in our group^32^ is meant to be an outlook for future applications and interesting fields of use.

**Fig. 1:**
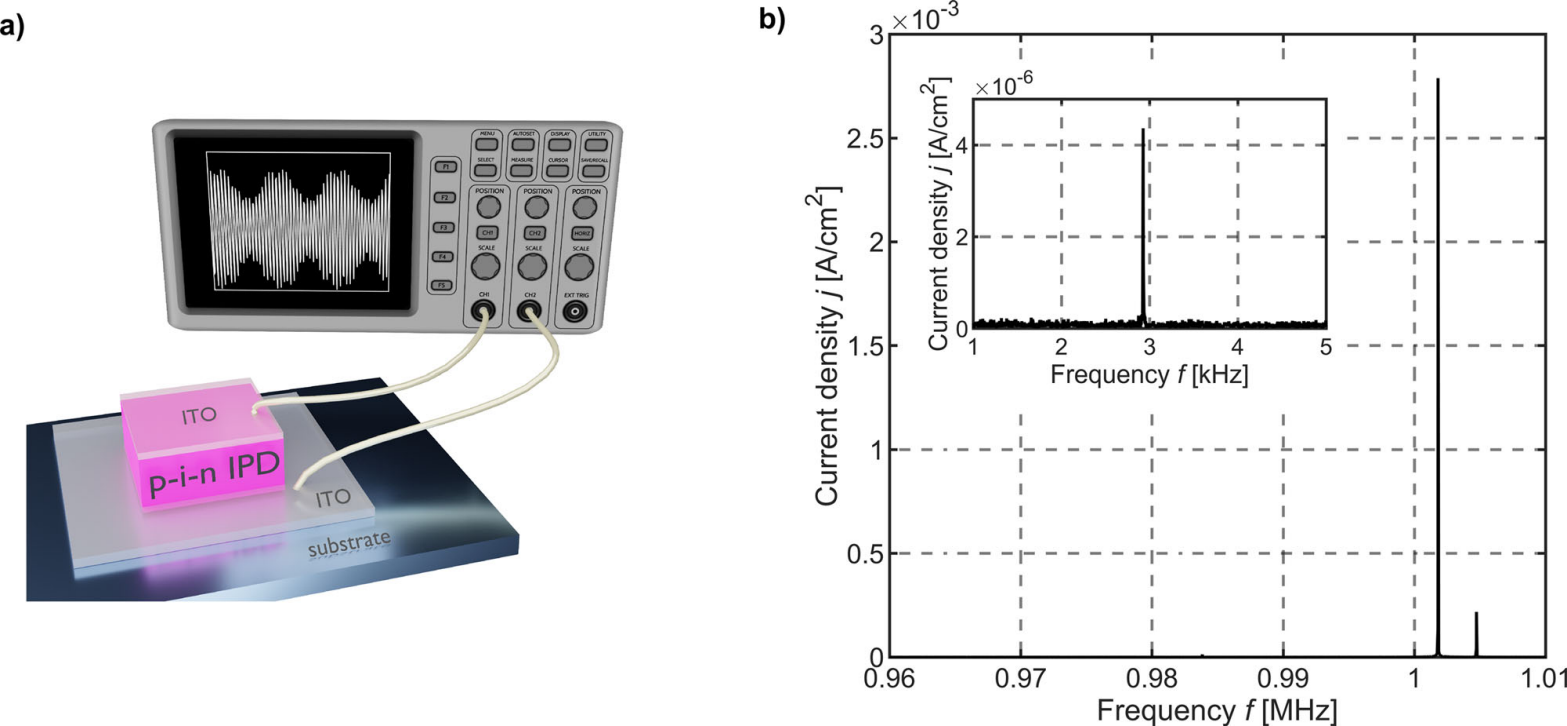
Device schematic and measurement. **a** Schematic of the Intrinsic Photomixing Detector (IPD) integrated on a glass substrate. **b** Measured fast Fourier transform of the IPD output current demonstrating envelope frequency mixing of two amplitude modulated light signals. The modulation signals of *λ*_*1*_ = 444 nm and λ_2_ = 636 nm are located at *f*_1_ = 1.004723 MHz and *f*_2_ = 1.001797 MHz, respectively. The inset shows the mixing frequency *f*_mix_ = *f*_*1*_ – *f*_*2*_ = 2.926 kHz that is directly generated by the IPD.

In this work, we present a promising device for direct and highly sensitive dual-wavelength envelope mixing, the Intrinsic Photomixing Detector (IPD), based on amorphous silicon (cf. Fig. 1a). As an application example, we demonstrate the capability to use an IDP as a ToF sensor for optical ranging and distance determination and discuss measurement results supported by electro-optical simulations. Compared to conventional optical ranging approaches, we exploit the photogating effect (cf. Supplementary Methods 2) in a simple amorphous silicon p-i-n photodetector that enables MHz bandwidth and allows for highly sensitive optical ranging at low light levels.

## Results and discussion

### Intrinsic photomixing in a-Si:H

Prior to optical ranging results, an initial experiment that demonstrates intrinsic dual-wavelength envelope frequency mixing (the “Intrinsic Photomixing”) in an a-Si:H IPD is presented serving as a prerequisite for follow-up ToF result studies and discussions. In general, the number of photogenerated electrons that might contribute to a photocurrent in a-Si:H p-i-n photodetectors is low for monochromatic blue wavelength illumination. This behavior is a result of a photo-induced local low-field region in the front of the device for short wavelength radiation. However, in case of dichromatic or multispectral illumination, the local low-field region might be offset so that the transport of electrons generated in the device front is facilitated. In result, a significantly larger amount of photogenerated electrons can drift towards the electrical contact so that collection efficiencies can exceed unity gain^27,29,33^. Such light-induced electrical field deformations enabled by photogating predominantly occur in defective materials, like a-Si:H, which exhibits a large amount of deep dangling bond states rather than localized tail states. In the following, dual-wavelength electrical field screening will be exploited for direct and highly sensitive envelope frequency mixing in amorphous silicon photodetectors at modulation frequencies exceeding MHz. A schematic of the IPD concept that enables ToF optical ranging is given in Fig. 1a. The IPD consists of an a-Si:H p-i-n thin-film multilayer stack sandwiched between two ~100 nm thin transparent conductive ITO (indium tin oxide) electrodes. The IPD prototype exhibits a total thickness of 1.5 μm and a photo-sensitive area of $A = 2.88\, {{{\rm{mm}}}}^2$ (1.6 mm × 1.8 mm), respectively. The sensor has been integrated on top of a glass substrate and patterned using conventional UV lithography. Further details on the low-temperature and CMOS compatible IPD sensor technology and process parameters are given in^34^, the Methods section and in the Supplementary Information (cf. Supplementary Methods 1).

In the experiment, two monochromatic light sources obtaining wavelength of *λ*_1_ = 444 nm and *λ*_2_ = 636 nm have been modulated with a sine stimulus and modulation frequencies of *f*_1_ = 1.004723 MHz and *f*_2_ = 1.001797 MHz. To conduct the upcoming experiments within the quasi-static limit of the device, optical modulation frequencies have been set far below the IPD cut-off frequency of ${f}_{c}\approx 2.2\,{{{{{\rm{MHz}}}}}}$. Choosing such modulations results in a frequency difference of ${f}_{{{{{\rm{mix}}}}}}={f}_{1}-{f}_{2}=2.926\,{{{{{\rm{kHz}}}}}}$, that is generated by the IPD itself (cf. inset Fig. 1b). The irradiance levels here are ${{{{{{\rm{\phi }}}}}}}_{\lambda 1}=10.2\,{{{{{\rm{mW}}}}}}{{{{{\rm{cm}}}}}}^{-2}$ and ${{{{{{\rm{\phi }}}}}}}_{\lambda 2}=5.7\,{{{{{\rm{mW}}}}}}{{{{{\rm{cm}}}}}}^{-2}$, respectively. The signal-to-noise ratio (SNR) on the nonlinear mixing frequency signal of SNR_mix_ = 37.5 dB indicates

1. the high envelope frequency mixing efficiency and sensitivity of the intrinsic photomixing in a-Si:H and
2. that irradiance levels can further be reduced in future experiments.

The SNR associated to the blue wavelength illumination is ${{{\rm{SNR}}}}_{444\,{{{\rm{nm}}}}} = 69.74\,{{{\rm{dB}}}}$, the corresponding SNR caused by red wavelength illumination is ${{{\rm{SNR}}}}_{636\,{{{\rm{nm}}}}} = 81.7\,{{{\rm{dB}}}}$, respectively.

Conventional crystalline photodetectors (e.g., Hamamatsu S1337-66BQ) did not show any mixing of envelope intensities at these comparatively low irradiance levels. Significantly higher irradiances would be required to enable an intrinsic nonlinear frequency conversion^35–38^ due to the inversion symmetrical crystallographic material composition.

### Optical ranging

In this section, experimental results on distance measurements exploiting intrinsic envelope frequency mixing of the IPD in combination with the indirect ToF principle are presented, evaluated and discussed. IPD ranging results demonste high sensitivities, the simplicity of the approach and capabilities for further optimizations. Compared to the direct ToF approach, where the round-trip Time-of-Flight is measured directly, we evaluate the phase shift of the mixing frequency rather than the round-trip time.

Direct ToF distance measurements rely on the finite speed of light propagation and the resulting time delay between emitted and received light. Depending on the specific implementation, modulated light or a light pulse is emitted, reflected by a scene, detected, and evaluated by receiver electronics with a time delay. Exemplarily, a depth resolution of one millimeter implies a temporal measurement resolution of 6.6 ps. To achieve such precisions, direct ToF sensors require highly accurate modulation and detection circuitry that are cost-intensive, complex and severely limit integration into miniaturized systems. Following the IPD concept, this integration effort can potentially be reduced by measuring phase shifts at comparable low frequencies in the kHz regime.

**Fig. 2:**
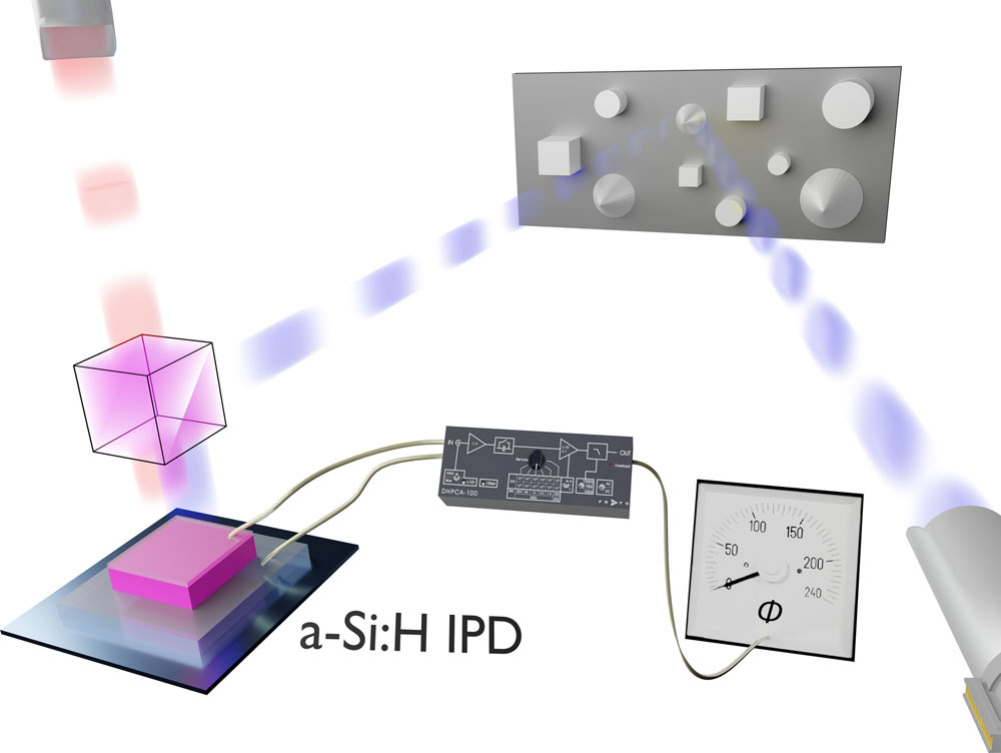
Scheme of the Time-of-Flight distance measurement setup utilizing an amorphous silicon- based Intrinsic Photomixing Detector. Modulated blue light is used for optical ranging in combination with a second red wavelength illumination source that remains at a fixed position relative towards the detector. The detector enables direct envelope frequency mixing of two modulated optical signals.

A schematic of the Time-of-Flight distance measurement setup using an a-Si:H IPD is given in Fig. 2. The light sources operate at wavelength of $\lambda_1 = 444\,{{{\rm{nm}}}}$ and *λ*_2_ = 636 nm with associated modulation frequencies of $f_1 = 1.004723\,{{{\rm{MHz}}}}$ and $f_2 = 1.001797\,{{{\rm{MHz}}}}$, respectively. The blue light is used for scene illumination, here represented by a highly reflective object (target reflectivity $\sim \!90 \%$), to evaluate achievable distance detection limits. The red illumination serves as the optical reference that is guided directly onto a dichroitic mirror (cf. Fig. 2) and remains at a fixed position relative to the IPD. The optical reflection of a spot or a scene is merged with the red illumination and guided onto the detector. The a-Si:H IPD operates at a fixed bias voltage of 0 V to achieve an optimized internal electric field screening at modulated dichromatic illumination conditions.

In the fabrication process, deposition parameters have been tuned and optimized in such a way, that a significant huge number of deep defect states occurs within the absorber material enabling envelope frequency mixing without external bias by preserving high photocurrents and keeping recombination rates moderate. Simulations on the influence of defects on the envelope mixing process and its efficiency have been published recently by Müller et al.^39^. Based on the simulation results, deposition parameters like the plasma power and gas mixture of the IPD have been tailored and optimized so that the absorber exhibits an optimized amount of deep dangling bond states.

Further details on fabrication parameters and measurement procedures are given in the Methods section.

Distance measurement experiments have been conducted by analyzing the range-dependent phase of the detector signal ${\varphi }_{{ToF}}(d)$ representing the Time-of-Flight of light from the blue light source to an object and back. The detector itself mixes this incoming light intensity with the intensity of the second red wavelength light source to a frequency component ${f}_{{{{{\rm{mix}}}}}}={f}_{1}-{f}_{2}$ with the corresponding phase ${\varphi }_{{f}_{{mix}}}={\varphi }_{{f}_{1}}-{\varphi }_{{f}_{2}}$. In the measurement, a zero adjustment of ${\varphi }_{{f}_{{mix}}}$ has been performed at the position $d=0$ which is the minimum distance between the scene and the face of the dichroitic mirror (here ~10 cm). In the following, this position serves as a reference calibration spot of the system as well as for distance zero adjustment by referencing the measurement results to ${\varphi }_{{ToF},d=0}$. In the ideal case, the phase *φ* of the mixing frequency component is linearly proportional to the distance $d$ according to the relationship^40^:

1 $$d=-c\cdot \frac{{\varphi }_{{ToF}}\left(d\right)-{\varphi }_{{ToF},d=0}}{2\cdot 2\pi \cdot {f}_{1}}=-c\cdot \frac{{\varphi }_{{f}_{{mix}}}(d)-{\varphi }_{{f}_{{mix}}}(0)}{2\cdot 2\pi \cdot {f}_{1}}$$

Equation (1) implies that by increasing the distance *d* between the detector unit and an object, the phase of *f*_*mix*_ is supposed to decrease linearly. Here, *c* represents the speed of light and *f*_1_ the modulation frequency of the blue wavelength illumination. Besides the achievable operating range *d*, a key parameter to evaluate the performance of an optical ranging system is the achievable depth resolution Δ*d*.

3D imaging systems that rely on the indirect ToF principle exploit measuring the phase shift of incoming light so that the standard deviation of the phase signal Δ*φ* corresponds to the achievable depth resolution Δ*d*^41^. That key figure of merit requires (i) evaluating a statistic significant number of measurements $n$ (here $n > 100$) at a fixed distance and (ii) approximating these results with a Gaussian distribution. In this specific experiment, the time for data acquisition to investigate achievable depth resolution limits is ~2 min. and mainly determined by the delay sequences between individual measurements. Data acquisition times can drastically be reduced, e.g. by taking less data points per measurement into account.

Reducing data points goes along with accuracy degradation that can be compensated for example by increasing signal-to-noise ratios or increasing electrical bandwidth since 1/*f* noise can be reduced significantly in a-Si:H p-i-n photodetectors^42^. The achievable accuracy in a future ranging application will strongly depend on the measurement realization, e.g. bandpass filter quality/steepness, integration time and noise of read-out electronics. Increased sensitivities will also lead to a more reliable and stable system with less deviation of measurement results and therefore higher accuracy.

**Fig. 3:**
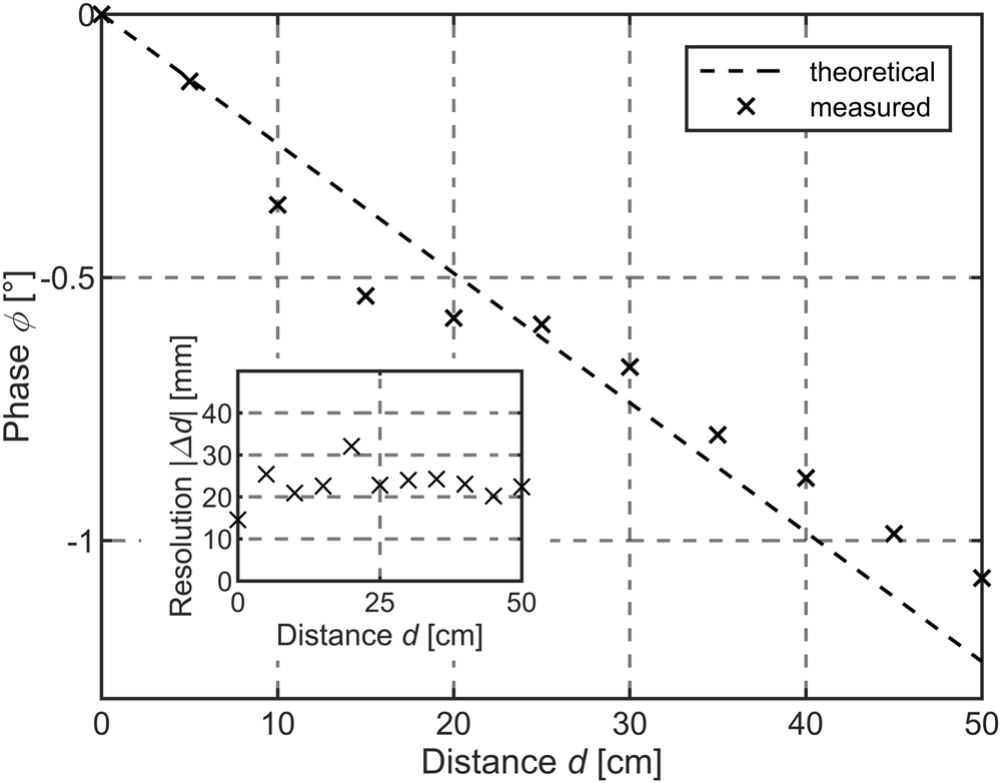
Short-range distance measurement. Theoretical (dashed) and experimentally measured phase signal (crosses) of the differential frequency component located at the frequency position *f*_mix_ = 2.926 kHz corresponding to a specific distance *d* according to Eq. (1). The inset shows the standard deviation of the phase signal resulting in a depth resolution down to *Δd* ≈ 14.5 mm at the reference distance *d* = 0 cm. The intrinsic photomixing detector achieves a mean depth resolution of *Δd* ≈ 22.9 mm below 50 cm.

**Fig. 4:**
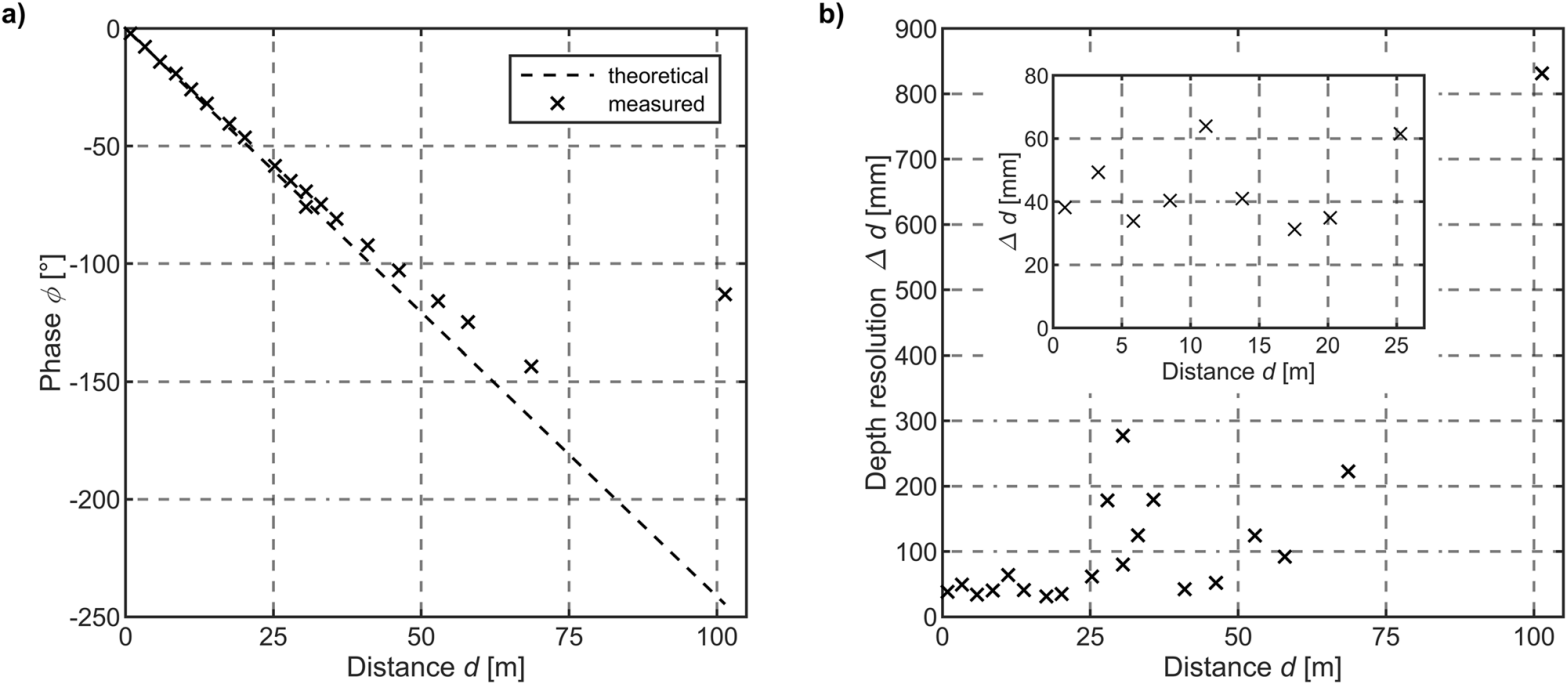
Long-range distance measurement. **a** Theoretical and experimentally determined phase shift of the mixing frequency component for varying distances. **b** Corresponding range-dependent depth resolution (${{{{\equiv }}}}$ standard deviation) of the distance measurement. The Intrinsic Photomixing Detector enables reliable distance measurements up to at least 100 m and a mean depth resolution of *Δd* ≈ 43.8 mm below 25 m. The depth resolution decreases at higher distances due to a gradual increase of the irradiance ratio on the sensor surface.

The standard deviation $\Delta \varphi$ can be expressed in degree (°) and directly be extracted from the lock-in amplifier or converted to a distance (mm) considering Equation (1). In the following experiments, the standard deviation $\Delta \varphi\;\triangleq\;\Delta d$ (here in mm) has been measured simultaneously at each specific distance to evaluate the achievable distance dependent depth resolution for a broad ranging bandwidth (cf. insets Figs. 3 and 4).

At first, the short-range distance measurement performance of the IPD has been investigated up to $d\le 50\,{{{{{\rm{cm}}}}}}$ prior to long-range studies for distances exceeding *d* > 100 m. Figure 3 shows the theoretical and measured phase shift of *f*_mix_ as a function of distance for the short-range scenario below 50 cm. The distance measurement results are in very good agreement with theory since the extracted phase of the mixing frequency $\varphi (d)$ decreases almost linearly by increasing the distance *d* (cf. Equation (1)). At each distance spot, the standard deviation has been calculated resulting in a mean depth resolution of $\Delta d\approx 22.9\,{{{{{\rm{mm}}}}}}$ for $0\,{{{{{\rm{cm}}}}}}\le d\le 50\,{{{{{\rm{cm}}}}}}$.

The optical irradiance for the red wavelength illumination remains constant at ${{{{{{\rm{\phi }}}}}}}_{\lambda 2}=5.7\,{{{{{\rm{mWcm}}}}}}^{-2}$ with a blue wavelength irradiance of ${{{{{{\rm{\phi }}}}}}}_{\lambda 1}=10.2\,{{{{{\rm{mWcm}}}}}}^{-2}$, respectively. The blue light intensity might slightly vary due to coupling losses into the measuring unit, defocusing of the beam and slight intensity variations within that distance range. The results not only demonstrate the ToF functionality, but optical ranging enabled by direct optical envelope frequency mixing within an a-Si:H IPD as an application example. The experiment verifies that the depth resolution of the IPD at distances $d\le 50\,{{{{{\rm{cm}}}}}}$ remains almost constant at such light levels and operating conditions.

To demonstrate the applicability of the nonlinear envelope intensity mixing process at distances exceeding 50 cm, further experiments have been conducted at mid- and long-ranges up to more than $100\,{{{{{\rm{m}}}}}}$. In contrast to short-range experiments, mid- and long-range measurements have not been performed in the laboratory but in the field on an overcast summer day in North Rhine-Westphalia (Germany) with ambient light of $\sim \!\!0.01\,{{{{{\rm{sun}}}}}}$ and without performing additional stray-light rejection to demonstrate the robustness of the principle. To guarantee reliable and comparable results and datasets, outdoor measurements have been performed within a specific time frame (stray light = constant) that limits the number of distance measurements exceeding $50\,{{{{{\rm{m}}}}}}$ presented in this work. Further experiments will evaluate and quantify suppression of backlight illumination capabilities and concentrate on acquiring more date points and optimizing performance, especially at long-range distances.

The results of the outdoor experiment are given in Fig. 4a, the corresponding range-dependent depth resolutions are shown in Fig. 4b. The measurements indicate that at short- and mid-range distances up to $d < \approx 25\,{{{{{\rm{m}}}}}}$, theory and experiment are in very good agreement. From the data, an almost constant mean depth resolution of $\Delta d \approx 43.8\,{{{\rm{mm}}}}$ below $d < \approx 25\,{{{\rm{m}}}}$ (cf. inset Fig. 4b) can be extracted corresponding to a relative distance deviation of $\frac{\triangle d}{d}=0.1752 \%$ in relation to the maximum range. Although mean depth resolutions slightly surpass short-range values ($\triangle d\approx 22.9\,{mm}$, cf. Fig. 3) the relative distance deviation is significantly reduced $\left(\frac{\triangle d}{d}=4.58 \% {;d}\le 50\,{{{{{\rm{cm}}}}}}\right)$. At longer distances, a progressive drift of the measured differential frequency phase in comparison to the expected phase value can be investigated (cf. Fig. 4a).

At *d* ≈ 18 m, the absolute phase difference between theory and measurement is $1.91^\circ$ that corresponds to a distance deviation of 75 cm. At such distances, the ratio of incoming photons that are reflected by a scene, or in this case a spot, to the constant amount of incoming photons emitted by the red wavelength ${\left.\vartheta =\frac{{{{{{{\rm{\phi }}}}}}}_{\lambda 1}}{{{{{{{\rm{\phi }}}}}}}_{\lambda 2}}\right|}_{d\approx 18m}\approx \frac{5\,{{{{{\rm{mWcm}}}}}}^{-2}}{3\,{{{{{\rm{mWcm}}}}}}^{-2}}=1.6\bar{6}$ is significantly reduced compared to the initial state at short ranges $\vartheta ={\left.\frac{{{{{{{\rm{\phi }}}}}}}_{\lambda 1}}{{{{{{{\rm{\phi }}}}}}}_{\lambda 2}}\right|}_{d\approx 0.8m}\approx \frac{30\,{{{{{\rm{mWcm}}}}}}^{-2}}{3\,{{{{{\rm{mWcm}}}}}}^{-2}}=10$. At distances exceeding $d > 100\,{{{{{\rm{m}}}}}}$, the phase shift between theory and measurement is drastically intensified since the amount of incoming photons further drops so that the irradiance ratio linearly deceases to ${\left.\frac{{{{{{{\rm{\phi }}}}}}}_{\lambda 1}}{{{{{{{\rm{\phi }}}}}}}_{\lambda 2}}\right|}_{d\approx 101m}\approx \frac{0.1\,{{{{{\rm{mWcm}}}}}}^{-2}}{3\,{{{{{\rm{mWcm}}}}}}^{-2}}=0.03\bar{3}$. However, in the experiment the lock-in amplifier reliably detects a mixing frequency component generated by the IPD at $d \approx 101\,{{{\rm{m}}}}$ and ${{{{{{\rm{\phi }}}}}}}_{\lambda 1}=0.1\,{{{{{\rm{mWcm}}}}}}^{-2}$ demonstrating (i) long-range distance measurement capabilities and (ii) the high sensitivity of the device enabled by photogating. To investigate the origin of the gradual increase of the phase shift at higher distances, electro-optical simulations have been performed. The results are presented in the following.

### Discussion of the phase deviation

To verify the hypothesis that a progressive phase drift at higher distances takes place due to significantly diverging intensities of blue and red wavelength on the sensor, electro-optical simulations have been performed. Therefore, a comprehensive simulation model of dichromatic modulation current measurements for an a-Si:H p-i-n IPD has been developed utilizing the software AFORS-HET^43^. Further details on the simulation model are given in ref. ^34^ and the Methods section. This model enables establishing a consecutive understanding of charge carrier generation and transport processes within the detector for different illumination scenarios.

**Fig. 5:**
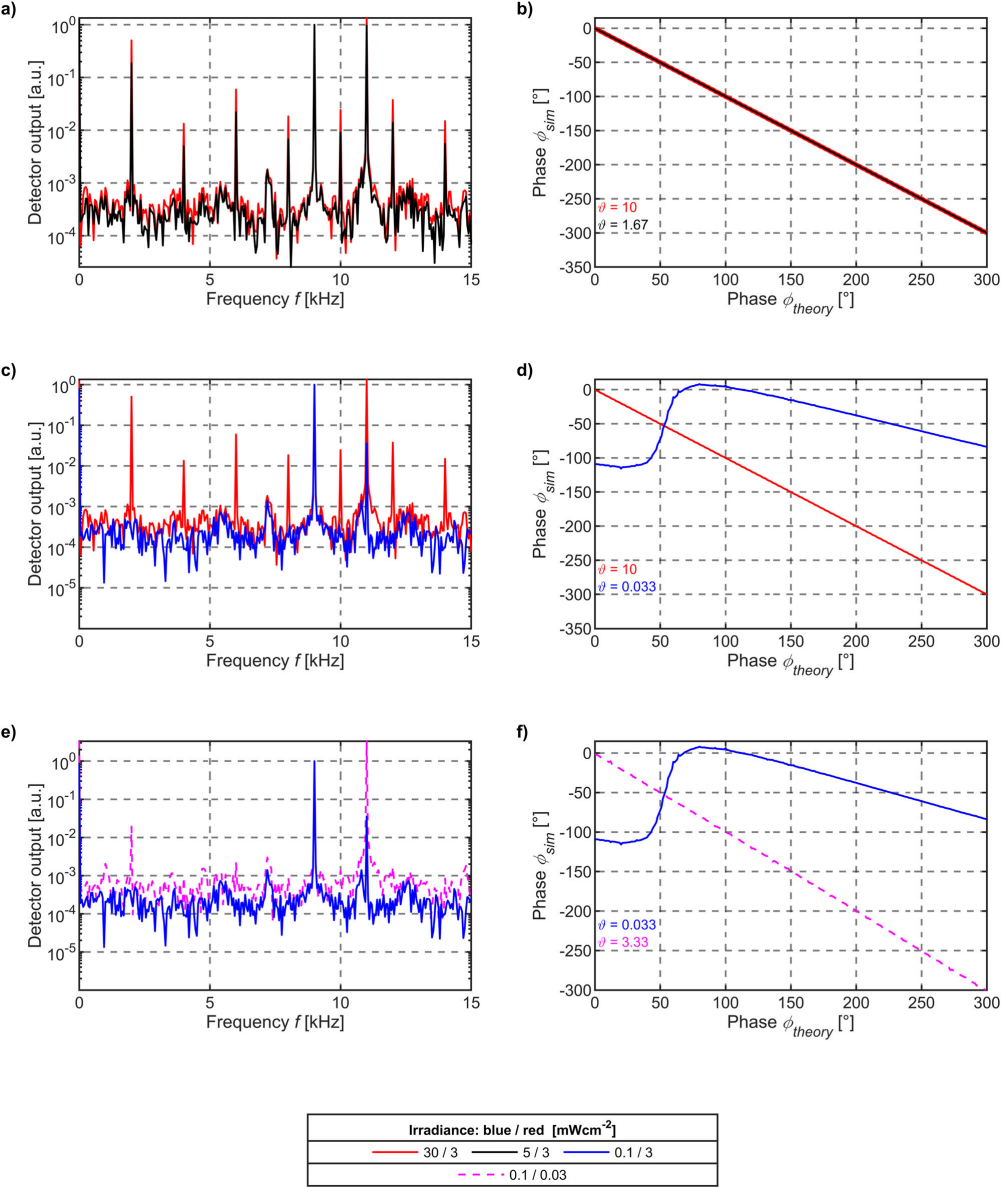
Simulation results of the phase deviation on the mixing frequency with varying irradiances. The modulation frequencies of blue and red light are at 9 kHz and 11 kHz, respectively. The phase simulation results are in very good agreement with theory for irradiance ratios ${{{{{{\boldsymbol{\vartheta }}}}}}\,{{{{{\boldsymbol{=}}}}}}\,{{{{{\boldsymbol{30}}}}}}}^{{{{{{\boldsymbol{x}}}}}}}$ with $\left|{{{{{\boldsymbol{\pm }}}}}}{{{{{\boldsymbol{x}}}}}}\right|{{{{{\boldsymbol{ < }}}}}}{{{{{\boldsymbol{1}}}}}}$.

In the simulation, dichromatic illumination with the wavelengths ${\lambda }_{1}=444\,{{{{{\rm{nm}}}}}}$ ($0.1\,{{{{{\rm{mWcm}}}}}}^{-2}{\le {{{{{\rm{\phi }}}}}}}_{\lambda 1}\le 30\,{{{{{\rm{mWcm}}}}}}^{-2}$) and ${\lambda }_{2}=636\,{{{{{\rm{nm}}}}}}$ (${{{{{{\rm{\phi }}}}}}}_{\lambda 2}={{{{{\rm{const}}}}}}.\approx 3\,{{{{{\rm{mWcm}}}}}}^{-2}$) have been modeled containing a rectangular stimuli. A consecutive sequence of quasi-static DC simulations has been merged enabling this light modulation scenario. The modulation frequencies of ${f}_{1}=11\,{{{{{\rm{kHz}}}}}}$ and ${f}_{2}=9\,{{{{{\rm{kHz}}}}}}$ have been chosen to be far below the IPD cut-off frequency of ${f}_{c}\approx 2.2\,{{{{\rm{{MHz}}}}}}$ to ensure that charge carrier dynamics remain within the quasi-static limit. To achieve a reliable distance measurement model based on the indirect ToF principle—more precisely the phase shift of *φ*(*d*)—a distinct time delay of the blue light modulation frequency with respect to the red stimulation has been implemented. Signal analysis has been conducted by evaluating the phase of the differential frequency signal as a function of the phase delay. Additionally, signal amplitude components in the frequency domain have been analyzed to evaluate the sensitivity of the internal mixing frequency process. In Fig. 5, electro-optical simulation results for varying blue wavelength irradiances are shown by keeping the peak irradiance of 3 mWcm^−2^ for red wavelength illumination constant. Such illumination cases exactly coincide with experimental optical ranging conditions presented and discussed in the previous section of this paper with varying range-dependent blue light intensities.

Figure 5a shows the fast Fourier transform (FFT) of the IPD output signal. The results reveal that a stable mixing frequency process within the IPD takes place at comparable irradiances, indicated by a significant signal amplitude located at the frequency position ${f}_{{{{{\rm{mix}}}}}}=2\,{{{{{\rm{kHz}}}}}}$. Here, the irradiance ratio of $\vartheta =\frac{{{{{{{\rm{\phi }}}}}}}_{\lambda 1}}{{{{{{{\rm{\phi }}}}}}}_{\lambda 2}}=\frac{30\,{{{{{\rm{mWcm}}}}}}^{-2}}{3\,{{{{{\rm{mWcm}}}}}}^{-2}}=10$ corresponds to the illumination scenario at short ranges of $d\approx 80\,{{{{{\rm{cm}}}}}}$ in the experiment. At such irradiance ratios, the simulated phase *φ*_sim_ is in perfect agreement with the expected ToF representative *φ*_theory_ (cf. Fig. 5b). The SNR on the mixing frequency component is ${{{{{\rm{SNR}}}}}}_{{f}_{{{{{\rm{mix}}}}}}}=62.16\,{{{{{\rm{dB}}}}}}$, respectively. In a next step, the blue wavelength intensity has been decreased by about two orders of magnitude (cf. Fig. 5c) compared to the initial simulation state and irradiance ratios of $\vartheta =10$ (cf. Fig. 5a, b). The result is an irradiance ratio of $\vartheta =\frac{{{{{{{\rm{\phi }}}}}}}_{\lambda 1}}{{{{{{{\rm{\phi }}}}}}}_{\lambda 2}}=\frac{0.1\,{{{{{\rm{mWcm}}}}}}^{-2}}{3\,{{{{{\rm{mWcm}}}}}}^{-2}}\approx 0.033$. This illumination case now depicts the distance measurement experiment at $d\approx 101\,{{{{{\rm{m}}}}}}$ (cf. Fig. 4a). Once the irradiance levels gradually diverge, a progressive phase deviation from the theory becomes visible in the electro-optical simulation (cf. Fig. 5d, $\vartheta =0.033$). That finding agrees with the measurement results presented in Fig. 4a at increasing distances. The FFT results verify that the signal component which is supposed to be located at the mixing frequency position *f*_*mix*_ vanishes in the noise floor. The strongly reduced signal-to-noise ratio of ${{{{{\rm{SNR}}}}}}_{{f}_{{{{{\rm{mix}}}}}}}=0.267\,{{{{{\rm{dB}}}}}}$ accordingly leads to nonlinear phase shift uncertainties (cf. Fig. 5d). The simulation results reveal that the mixing process becomes increasingly unstable at an irradiance ratio exceeding $\vartheta ={30}^{x}$ with $\left|\pm x\right| > 1$. Conclusively, the quality of the mixing process and thus the stability of the phase shift (cf. Supplementary Note 1) in ongoing experiments can be reinforced, e.g. by a real-time adaptive control of the red wavelength irradiance on the detector. To verify that hypothesis, an additional simulation has been conducted by further decreasing the red irradiance so that both, blue and red illumination intensities, once again become comparable but at absolute lower light levels below mWcm^−2^. For irradiance ratios of $\vartheta =\frac{{{{{{{\rm{\phi }}}}}}}_{\lambda 1}}{{{{{{{\rm{\phi }}}}}}}_{\lambda 2}}=\frac{0.1\,{{{{{\rm{mWcm}}}}}}^{-2}}{0.03\,{{{{{\rm{mWcm}}}}}}^{-2}}\approx 3.33$, the mixing frequency component is lifted out of the noise floor in the FFT with a ${{{{{\rm{SNR}}}}}}_{{f}_{{{{{\rm{mix}}}}}}}=30.87\,{{{{{\rm{dB}}}}}}$ (cf. Fig. 5e) so that the simulated phase *φ*_sim_ again is in perfect agreement with the expected ToF representative *φ*_theory_ (cf. Fig. 5f).

Table 1 summarizes figures of merit of state-of-the-art solid-state ToF 3D imagers and LiDAR systems and the results achieved in this work including detector type, illumination source and wavelength, operating range and depth resolution. A fair comparison between 3D imaging sensors and systems is not possible since there is no standardized measurement scenario, i.e. target reflectivity and ambient light might differ significantly. However, and since these values are typically highlighted in datasheets of commercial range finders to classify performance, target reflectivity and stray-light rejection have been included.

**Table 1:** IPD figures of merit and comparison with state-of-the-art solid-state Time-of-Flight 3D imagers and LiDAR systems.

Sensor module/detector/principle	Wavelength *λ*/illumination source	Operating range/distance *d*	Depth resolution $\varDelta d$	Annotations
a-Si:H p-i-n intrinsic photomixing detector (ToF)	*λ*_1_ = 444 nm *λ*_2_ = 636 nm (Solid-state laser)	0 < *d* < 50 cm	22.9 mm	This work* *Φ*_*1*_ = 30 mW/cm^2^ @ 90% target reflectivity ~1 klux ambient light immunity Single pixel
		1 m < *d* < ≈25 m	43.8 mm	This work* *Φ*_1_ = 5 mW/cm^2^ @ 90% target reflectivity ~1 klux ambient light immunity Single pixel
		d ≈ 101.37 m	0.81 m	This work* Limit of detection *Φ*_1_ = 0.1 mW/cm^2^ @ 90% target reflectivity ~1 klux ambient light immunity Single pixel
SPAD Camera ^15^(Time-gated ToF)	637 nm (Solid-state laser)	0.2 m < d < 1.6 m	7.8 mm	1.6 m @ ~60% target reflectivity 1024 × 500 pixel (FF: 13.4%)
Active Meta-surface SLM + APD ^18^(LiDAR)	1560 nm (Solid-state laser)	*d* ≤ 10 m	4 cm	85 W peak/85 mW average power on device 550 nano-resonators 16 × 5 APD pixel
Benewake TF03-180 (LiDAR)**	905 nm (Laser diode)	0.1 m < *d* < 180 m	< 30 mm	180 m @ 90% target reflectivity 0.1 m–130 m @ 90% target reflectivity 100 klux ambient light immunity
Ifm 03D303 Photonic mixing device (CW-ToF)**	850 nm (LED)	30 cm < *d* < 1 m	8 mm	@18% reflectivity (gray object) 8klux ambient light immunity 352 × 264 pixel
		7 m < *d* < 8 m	50 mm	
ams-OSRAM TMF8820 SPAD (Direct ToF)**	940 nm (VCSEL)	1 cm < *d* < 5 m	1 mm + (*d* ∙ 0.25%)	@18% target reflectivity (gray object) 350 lux (LED) ambient light immunity 18 × 18 SPAD pixel
Terabee TeraRanger Evo 60 m Opto-electronic sensing device (ToF)**	940 nm (High-power LED)	0.5 m < *d* < 14 m	±4 cm	60 m @ white target/no sunlight sunshine/low reflectivity targets (e.g., grass) can reduce max. range to *d* < 10 m
		14 m <*d* < 60 m	±1.5%	
Terabee TeraRanger Evo 3 m Opto-electronic sensing device (ToF)**	940 nm (High-power LED)	0.1 m < *d* < 3 m	±2 cm	3 m @ white wall/medium NIR ambient halogen light (indoor) Wood/cardboard target detection without NIR ambient light (indoor)
Fotonic E70 CCD (CW-TOF)**	850 nm (High-power LED)	0.1 m < *d* < 10 m	5 mm @ 0.15–1 m (E70 4 W)	@ 70% target reflectivity 160 × 120 pixel 100 klux ambient light immunity
			30 mm @ 5–7.5 m (E70 16 W)	
LUCID Helios2 CMOS Sony DepthSense IMX556PLR (ToF)**	850 nm (VCSEL)	0.3 m < *d* < 8.3 m	0.8 mm @ 0.5 m	White paper target 640 × 480 Pixel On-chip ambient light filter
			14.48 mm @ 8.3 m	

The benchmarks include illumination source and wavelength, operating range and depth resolution.

*Measured @ fixed bias irradiance of Φ_2_ = 3 mW/cm^2^; no additional data post-processing performed.

**Commercially available.

Compared to other sensors, PMDs integrate an additional SBI (Suppression of Background Illumination) circuit on-chip to achieve very good ambient light immunity of $\sim \!\!150\,{{{{{\rm{klux}}}}}}$^44^. ToF and LiDAR sensors also integrate microlenses to achieve high sensitivities. Without any stray-light rejection in terms of additional circuitry or optical bandpass filtering and without microlens integration, the IPD approach presented in this work already achieves $\sim \!\!1\,{{{{{\rm{klux}}}}}}$ ambient light immunity that was given by the measurement environment in the field on an overcast summer day in North Rhine-Westphalia (Germany). However, further experiments will include investigations on stray-light rejection capabilities.

The IPD already achieves detection limits down to at least 0.1 mWcm^−2^ at blue wavelength enabled by photogating. In ref. ^45^, IPD sensitivities of 744 mAW^−1^ have been reported at blue wavelength enabled by photogating with ${\lambda }_{2}=636\,{{{{{\rm{nm}}}}}}$ as used in this work. That sensitivity depends on the gating irradiance and wavelength and can exceed benchmarks of conventional crystalline silicon p-n or p-i-n junction photodetectors (e.g. 243 mAW^−1^ @ 477 nm for the Hamamatsu S1337-66BQ used as reference detector) demonstrating the huge potentials of this approach for manifold applications. Optimizing the sensor structure, layer thicknesses and defect density distributions of the material in conjunction with the photogating scenario allows to maximize the nonlinear field screening that can enable further performance improvements. Besides, sensitivities might be increased further by integrating anti-reflection coatings (reflectivity of the sensor: $\approx 42 \% \,@\,444\,{{{{{\rm{nm}}}}}}$, $\approx 9 \% \,@\,636\,{{{{{\rm{nm}}}}}}$; cf. Supplementary Data 1) or back reflectors to enhance internal quantum efficiencies. That comparatively high reflectivity $@\,444\,{{{{{\rm{nm}}}}}}$ can mainly be attributed to standing waves within the ITO due to the ITO/a-Si:H multilayer structure of the sensor.

## Conclusion

In this work, intrinsic envelope frequency mixing for dual-wavelength illumination in the visible range has been demonstrated in a very simple p-i-n photodetector structure out of amorphous silicon at low irradiance levels down to $\sim \!0.1\,{{{{{\rm{mWcm}}}}}}^{-2}$. The IPD approach has been demonstrated with modulation frequencies exceeding MHz and 0 V bias voltage. The device enables optical ranging for distances exceeding 101 m in first proof-of-concept demonstrations. At distances below 25 m, a mean depth resolution of 43.8 mm could be achieved which is already comparable to commercial solid-state ToF or LIDAR systems. In the short distance range below 50 cm, depth resolutions of ~23 mm could be achieved. Experimental studies revealed an interesting phenomenon, namely a progressive phase deviation taking place at larger distances. Electro-optical simulations verify an intensity induced phase shift once irradiance ratios of the light modulations surpass values of at least $\vartheta ={30}^{x}$ with $\left|\pm x\right| > 1$. The quality of the mixing process and thereby the phase stability can be reinforced, e.g., by a real-time adaptive control of the red wavelength irradiance on the detector, which has been verified by additional simulations. Compared to conventional ToF sensors, such as the widely established PMD, a-Si:H thin-film technology benefits from low fabrication temperatures below 200 °C and is highly compatible with silicon CMOS back-end integration or the integration into flexible electronics and wearables with geometrical fill factors up to 100%. Moreover, IPDs can be operated at zero bias resulting in low power consumption making the approach a power efficient, scalable and very sensitive detector alternative compared to conventional electro-optical ToF or LIDAR sensors. Since photogating in a-Si:H can enable collection efficiencies beyond unity gain, sensitivities might be increased further in future ranging or 3D imaging experiments.

## Methods

A-Si:H thin-films have been deposited by low-temperature PECVD below 200 °C in a hot-wall MVS multi-chamber deposition system on pre-cleaned glass substrates. Transparent and conductive ITO anode and cathode electrodes have been sputtered in a radio frequency hot-wall sputtering reactor at 13.56 MHz below 50 °C. The IPDs were patterned to 1.6 mm × 1.8 mm using standard UV-lithography prior to dicing, mounting the detectors in dual in-line chip carriers and final wedge bonding for contacting. The bonding has been conducted using a semi-automatic TPT HB16 wirebonder. Further fabrication details are given in the Supplementary Information (cf. Supplementary Methods 1) and in ref. ^46^. Thin-film growth rates of a-Si:H and ITO have been determined by cross-sectional back-scatter scanning electron microscopy (SEM) imaging using a FEI Quanta 250 environmental SEM. Total device thicknesses of a-Si:H IPDs have been validated using a Bruker Dektak XT profilometer.

Electro-optical simulations have been conducted using the simulation software AFORS-HET^43^ taking into account an appropriate device model. This model has been used to subsequently develop, fabricate, and characterize the field-optimized a-Si:H IPDs. Low-frequency transient simulations have been performed by a set of consecutive steady-state simulations in the time domain. A bandwidth of 100 ms has been discretized with a resolution of 990,001 time stamps. Rectangular modulation signals serve as input parameters for the illumination sources.

Time and frequency domain envelope frequency mixing measurements utilize a 444 nm Toptica iBEAM-SMART-PT-445CZ_20067 and a 636 nm Toptica iBEAM-SMART-636-S-KL-11049 light source. The lasers have been modulated using conventional function generator (TGA 1244). Transient and FFT signal acquisition has been realized with a Rhode&Schwarz HMO 3004 digital oscilloscope prior to an I-V conversion utilizing a DHPCA100 amplifier module (FEMTO Messtechnik GmbH). The cut-off frequency of the device has experimentally been determined by measuring the series capacitance and resistance of the IPD using a Hewlett Packard LCR-meter.

The experimental ranging measurement setup comprises a dichroic mirror, a lens, and an optic diffuser to ensure a homogeneous illumination irradiance on the sensor. The bias voltage of the device has been fixed to 0 V to eliminate external bias induced influences on the internal electric field and thereby the envelope frequency mixing process.

The acquisition of the amplitude and phase of the mixing frequency has been realized using a Princeton Applied Research 5210 Dual Phase Lock-in amplifier in combination with the same measurement equipment as for the time and frequency domain measurements.

Irradiances for long-range distance measurements have been determined in front of the dichroitic mirror using a Coherent power meter with an active area of 1 cm^2^.

### Supplementary information

Supplementary Information

## Data Availability

The data used in this paper is available on request from the corresponding author.
