# Supplementary material for: Amorphous silicon intrinsic photomixing detector for optical ranging
Source: Commun Eng. 2023 Dec 4;2:85. doi: 10.1038/s44172-023-00137-5 (PMC10955829; doi:10.1038/s44172-023-00137-5)
Supplement: Supplementary file 1 — Supplementary Information [file 44172_2023_137_MOESM1_ESM.pdf]

Supplementary Information

Amorphous Silicon Intrinsic Photomixing Detector for Optical Ranging

Andreas Bablich[1†*], Maurice Müller [1†], Rainer Borneman[1†], Nils Marrenbach[1], Paul Kienitz[1], Peter Haring Bolívar[1]

† These authors contributed equally

* Corresponding author: andreas.bablich@uni-siegen.de

[1] University of Siegen – School of Science and Technology

Hölderlinstr. 3, 57068 Siegen, NRW, Germany

Supplementary Methods 1

a-Si:H Technology and Processing

Schott D263 glass substrates (5 cm x 5 cm) with a high glass transition temperature and low surface roughness have been chosen for optimized multilayer thin-film deposition. Prior to the high vacuum deposition processes, the samples have been thoroughly cleaned in acetone and isopropanol for 3 minutes in an ultra-sonication bath (DI-water and N_2 drying after each step). PVD magnetron sputtering at 13.56 MHz has been used as the deposition technology to form indium tin oxide (ITO) contacts. Subsequent layers have been grown in a high vacuum MVS multi-chamber cluster-tool including four PE-CVD deposition chambers operating at a radio frequency of 13.56 MHz (cf. Figure S 1).

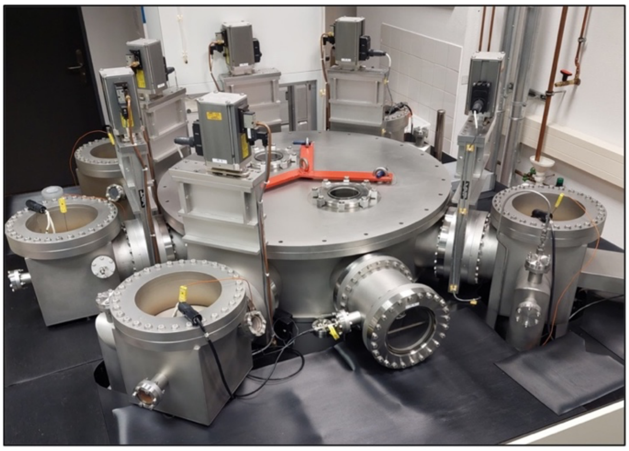

Figure S 1 Plasma-enhanced chemical vapor deposition multi-chamber cluster-tool for amorphous silicon thin-film deposition.

Silane (SiH_4) serves as a precursor for a-Si:H. Phosphine (PH_3) or diborane (B_2H_6) have been added to the SiH_4 gas mixture to realize n-/p-type a-Si:H. Detailed process parameters are given in Table S 1. The IPD devices have been patterned to 1.6 mm x 1.8 mm by conventional UV contact lithography using a conventional photoresist AZ5214E (Microchemicals GmbH). The ITO anode has been etched using hydrochloric acid (HCl) with a concentration of 5.5%. Reactive ion etching of a-Si:H has been realized using a SWAFER (Cobrain) system with 100 sccm SF_6 and 50 sccm Ar at 100 W and 0.133 mbar.

Table S 1 Deposition parameters of the thin-film layers of the Intrinsic Photomixing Detector.

a-Si:H / p-i-n photomixer	
cathode / thickness [nm]	ITO / ~100
eff. RF-power [W] / time [s]	100 / 120
process pressure [mbar]	0.055
temperature [C°]*	40
n-a-Si:H / thickness [nm]	~10
precursor: SiH_4 [sccm] / PH_3 [sccm]	20 / 15
eff. RF-power [W] / time [s]	6.9 / 120
pressure [mbar]	0.667
temperature [C°]*	200
i-a-Si:H/ thickness [nm]	1,520
precursor: SiH_4 [sccm]	20
eff. RF-power [W] / time [s]	7.1 / 3000
process pressure [mbar]	0.667
temperature [C°]*	200
p-a-Si:H/ thickness [nm]	~10
precursor: SiH_4 [sccm] / B_2H_6 [sccm]	20 / 15
eff. RF-power [W] / time [s]	6.9 / 120
process pressure [mbar]	0.667
temperature [C°]*	200
anode/ thickness [nm]	ITO / ~100
RF-power [W] / time [s]	100 / 120
Pressure [mbar]	0.055
temperature [C°]*	40

* hot-wall reactor design: temperature at the process chamber wall

Supplementary Methods 2

j-V Characteristics

Basic device properties and diode functionality have been verified utilizing current density-voltage measurements under defined illumination conditions. The experiments have been conducted at room temperature with a Keithley 4200-SCS parameter analyzer in combination with a Suss microprobe station. RGB-LEDs have been used to determine the device response on blue, red and dichromatic illumination. The resulting measurements are given in Figure S 2 and reveal the expected conventional a-Si:H photodiode behavior. The influence of current quenching due to trap-induced electrical field screening is visible in the j-V characteristics of monochromatic blue illumination around 0 V bias. Also, the current increase at dual wavelength illumination is visible since the expected current density

$$j_{expected} = j_{blue}(V) + j_{red}(V) - j_{dark}(V)$$

drops below the actual measured value. This underlines the applicability of the device for the amplitude modulated optical frequency mixing presented in this work.

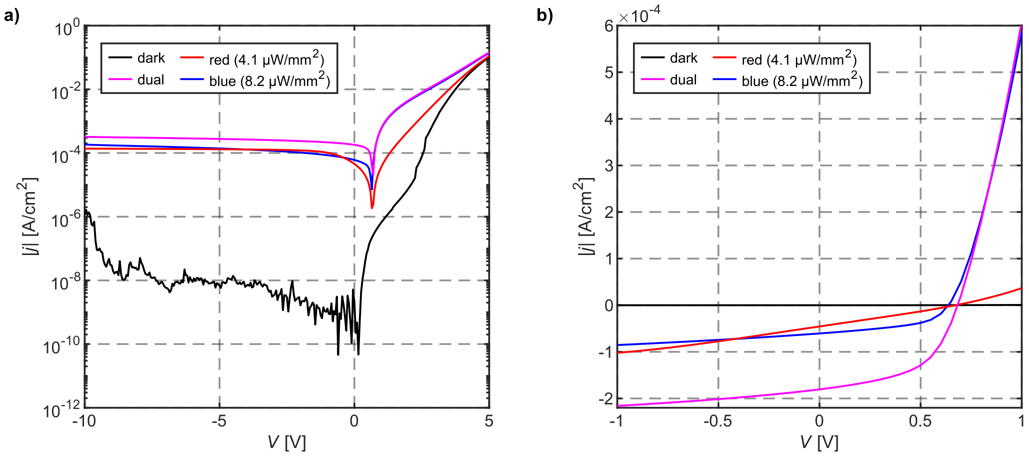

Figure S 2 Current density/voltage characteristics of the Intrinsic Photomixing Detector under different illumination scenarios. a) Logarithmic scale and b) close-up (linear scale) around 0 V bias to visualize the current quenching mechanism due to trap-induced electrical field screening.

Supplementary Note 1

Phase Stability

The accuracy at specific distances has been determined using the standard deviation of the measured phase signal. In Figure S 3, the measured phase signals are visualized exemplarily for a) a short range of $d = 0.86\,m$ and b) for the maximum distance covered in this work of $d = 101.37\,m$. In both cases, the phase displacement distribution with a total amount of $n = 200$ measurements can be modeled with a gaussian function and the corresponding standard deviations (≡ accuracy). The results illustrate the reliability of the IPD phase measurement approach.

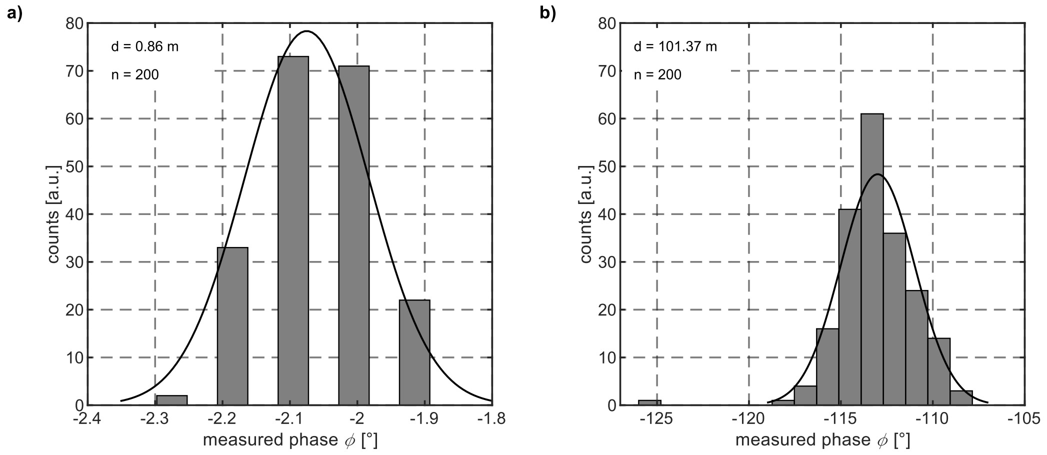

Figure S 3 Distribution of the measured phase signal for a fixed distance and a total amount of 200 measurements per distance. a) For a short-range of 0.86 m and b) for the maximum distance covered in this work of 101.37 m. Both results can be modeled using a gaussian distribution (solid lines) and the corresponding standard deviations.

Supplementary Data 1

Transmission/Reflection Spectra

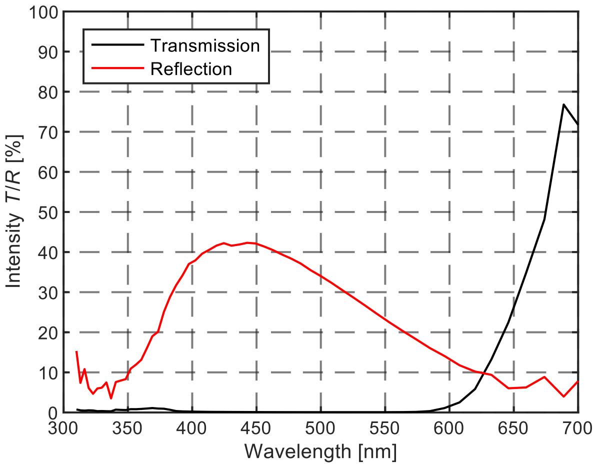

Figure S 4 Transmission and reflection spectra of the Intrinsic Photomixing Detector.
